# Serial mediation pathways linking attachment security to loneliness: the role of self-disclosure quality and perceived social support among college students

**DOI:** 10.3389/fpsyg.2026.1749281

**Published:** 2026-02-11

**Authors:** Hong Guo, Qian Zhu, Yikun Zhong, Wei Huang

**Affiliations:** 1 Training and Research Center of Ideological and Political Work Team of Ministry of Education, Shaanxi Normal University, Xi’an, China; 2 State Key Laboratory of Oil and Gas Reservoir Geology and Exploitation, Chengdu University of Technology, Chengdu, China; 3 Chengdu University of Traditional Chinese Medicine, Chengdu, China; 4 School of Marxism, Southwest Petroleum University, Chengdu, China; 5 College of Preschool and Primary Education, China West Normal University, Nanchong, China

**Keywords:** attachment security, college students, loneliness, perceived social support, serial mediation

## Abstract

**Background:**

Loneliness among college students has reached epidemic proportions, yet the specific behavioral and environmental mechanisms through which attachment security protects against loneliness remain unclear. While previous research has focused on how attachment insecurity contributes to loneliness, less attention has been paid to understanding the protective pathways of attachment security. This study examined a serial mediation model in which self-disclosure quality and perceived social support sequentially mediate the attachment security-loneliness relationship.

**Methods:**

A cross-sectional survey of 1,098 Chinese college students (M_age = 20.15, SD = 1.28; 82.9% female) assessed attachment security (closeness dimension), self-disclosure quality (depth and honesty composite), perceived social support, and loneliness using validated instruments. Serial mediation analysis was conducted using PROCESS Model 6 with bootstrap confidence intervals.

**Results:**

The model explained 43.2% of loneliness variance. Three significant indirect pathways emerged: perceived social support alone (B = −0.433, 69.5% of total indirect effect), complete serial mediation through self-disclosure quality and perceived social support (B = −0.110, 16.9%), and self-disclosure quality alone (B = −0.084, 13.6%). All hypothesized relationships were supported, with perceived social support serving as the dominant protective mechanism.

**Conclusion:**

Perceived social support represents the primary pathway linking attachment security to reduced loneliness, while behavioral mechanisms through self-disclosure quality provide secondary protection. These findings advance understanding of attachment security’s protective mechanisms and suggest that interventions should prioritize enhancing students’ capacity to recognize and utilize available social resources, with supplementary focus on developing high-quality disclosure behaviors.

## 1 Introduction

Loneliness is defined as the subjective, distressing experience that occurs when there is a discrepancy between desired and actual social relationships (Peplau and Perlman, 1982; Russell, 1996). Recent advances emphasize that loneliness requires a multidimensional approach, distinguishing between emotional loneliness (perceived lack of intimate connections) and social loneliness (perceived deficits in social networks), with these dimensions having distinct correlates and consequences (Walsh et al., 2025). This multifaceted phenomenon has emerged as a critical public health concern among college students, with prevalence rates reaching epidemic proportions and significant implications for academic performance, mental health, and long-term wellbeing (Holt-Lunstad et al., 2017). Large-scale longitudinal research involving over 128,000 participants across multiple countries reveals that loneliness follows a U-shaped trajectory across the lifespan, typically decreasing from young adulthood to midlife before increasing in older adulthood (Graham et al., 2024). Moreover, loneliness varies significantly across time and geographic contexts, influenced by macro-level factors including cultural values, social norms, and technological changes, highlighting its complex, socially embedded nature (Luhmann et al., 2023). A recent systematic review and meta-analysis found a significant increase in loneliness during the COVID-19 pandemic compared with pre-pandemic levels, reflecting a modest but consistent global rise in perceived social isolation (Ernst et al., 2022). While pandemic-related restrictions have largely been lifted, understanding the fundamental mechanisms that protect against loneliness remains crucial for promoting psychological adjustment among emerging adults. The college transition represents a particularly vulnerable period characterized by separation from established social networks, identity exploration challenges, and the need to develop new relationships in unfamiliar environments (Wuthrich et al., 2020). Understanding the mechanisms that protect against loneliness during this critical developmental period is essential for developing effective prevention and intervention strategies that can promote psychological adjustment and academic success among emerging adults (Ellard et al., 2023).

### 1.1 Attachment and loneliness: theoretical foundations and empirical evidence

Recent theoretical advances have provided nuanced understanding of the relationship between attachment processes and different forms of subjective disconnection. Contemporary frameworks distinguish between emotional loneliness (perceived absence of close or intimate relationships), social loneliness (perceived absence of an engaging broader social network), and existential isolation (perceived absence of shared perceptions and experiences) (Helm, 2025). These distinctions are important because different attachment orientations may relate differentially to specific forms of loneliness, with anxious attachment more strongly associated with emotional loneliness and avoidant attachment linked to social loneliness and existential isolation (Helm et al., 2020; Bergman et al., 2024).

Attachment theory provides a fundamental framework for understanding how early relational experiences shape individuals’ capacity for emotional regulation and interpersonal functioning throughout the lifespan (Bowlby, 1969). The closeness dimension of adult attachment represents a particularly robust indicator of attachment security, reflecting individuals’ comfort with emotional intimacy, capacity for trust, and willingness to depend on others in times of need (Collins and Read, 1990). This dimension captures positive internal working models of others and confidence in relationships as sources of safety and support, characteristics that facilitate the development and maintenance of meaningful social connections (Simpson and Rholes, 2017). Meta-analytic and large-scale correlational findings suggest that secure attachment serves as a protective factor against loneliness, whereas anxious and avoidant attachment styles are associated with heightened feelings of social isolation (Zhang et al., 2022). The college years represent a particularly critical developmental period for examining attachment-loneliness relationships, as emerging adults navigate complex tasks of identity formation and intimate relationship establishment while often separated from familiar social support systems (Arnett, 2000). However, the specific mechanisms through which attachment security influences loneliness experiences remain incompletely understood, necessitating investigation of the behavioral and environmental pathways that mediate these relationships.

### 1.2 Self-disclosure quality as relational behavior pathway

Self-disclosure quality, encompassing the depth and honesty of personal information sharing, represents a fundamental interpersonal process that facilitates relationship development and psychological wellbeing (Sprecher and Hendrick, 2004). Established theoretical frameworks emphasize the multidimensional nature of disclosure behaviors, recognizing that psychological and relational benefits depend more on qualitative characteristics than on mere quantity or frequency (Reis and Shaver, 1988). Contemporary research continues to provide robust empirical support for this perspective. A recent comprehensive meta-analysis of 38 studies found that the quality dimensions of self-disclosure—specifically valence and honesty—were moderately and positively associated with psychological wellbeing, while the quantity of self-disclosure showed no significant association with wellbeing outcomes (Chu et al., 2023). Similarly, research with adolescents demonstrates that deeper, more intimate self-disclosures are associated with better mental health and wellbeing compared to superficial disclosures (Nowell et al., 2023). Additional evidence indicates that the quality of online self-disclosure, rather than mere frequency, determines its impact on psychological adjustment and social relationships (Dhir et al., 2021). The depth dimension reflects the personal significance and emotional intensity of disclosed information, while the honesty dimension captures the accuracy and authenticity of shared content (Altman and Taylor, 1973; Wheeless, 1976). Contemporary evidence continues to validate these dimensions, with research showing that deeper disclosures are associated with higher relationship quality and wellbeing (Towner et al., 2022), while authentic, balanced self-disclosure enhances credibility and trustworthiness in interpersonal communications (Lee and Johnson, 2022).

Attachment theory provides a compelling framework for understanding individual differences in self-disclosure quality patterns. Individuals with higher attachment security, characterized by positive internal working models, are theoretically predicted to engage in higher-quality disclosure behaviors due to their greater comfort with vulnerability and trust in others’ responsiveness (Collins and Miller, 1994). Empirical research has consistently supported these predicted associations: across eight studies, securely attached individuals displayed greater authenticity and honesty in close relationships—and security priming further increased state authenticity while reducing lying and cheating—indicating a stronger tendency toward sincere self-disclosure compared with those higher in attachment anxiety or avoidance (Gillath et al., 2010).

The adaptive functions of high-quality self-disclosure operate through both intrapersonal and interpersonal mechanisms. At the intrapersonal level, authentic self-expression facilitates emotional processing and identity clarification (Pennebaker and Chung, 2011). At the interpersonal level, quality disclosure conveys trust and intimacy intentions, elicits reciprocal disclosure, and fosters mutual understanding, thereby strengthening social bonds and facilitating social support (Sprecher et al., 2013). Evidence indicates that greater depth and authenticity in self-disclosure are associated with lower loneliness, in part via enhanced perceived social support and social connectedness (Luo and Hancock, 2020).

### 1.3 Perceived social support as environmental response mechanism

Perceived social support represents a critical environmental resource that emerges from social interactions and significantly influences psychological adjustment outcomes. Cohen and Wills’ (1985) main effect model proposes that perceived social support directly enhances wellbeing regardless of stress levels. This model proves particularly relevant for understanding loneliness, as perceived support availability may directly counteract feelings of social isolation through its impact on belongingness perceptions. The process through which self-disclosure quality behaviors mobilize perceived social support represents a fundamental mechanism linking interpersonal behavior to environmental resources. High-quality self-disclosure serves multiple signal functions that facilitate supportive responses from relationship partners. These behaviors communicate trust, vulnerability, and openness to support and connection (Clark and Lemay, 2010). This disclosure-to-support mobilization process operates via both affective and informational pathways. Emotional disclosure yields downstream emotional, relational, and psychological benefits. It effectively conveys needs while eliciting empathic responses that motivate supportive behavior (Ho et al., 2018).

The relationship between perceived social support and loneliness has been extensively documented across diverse populations through meta-analytic evidence (Gariépy et al., 2016). College students face unique challenges that make perceived social support particularly relevant for loneliness prevention, as the transition to college often involves separation from established support networks and the need to develop new support systems. Students who access and utilize broader social support subsequently report less loneliness, which in turn buffers later adjustment difficulties (e.g., compulsive Internet use, time-management, interpersonal/health problems) during the college transition (Zhang et al., 2018).

### 1.4 Research gaps and current study

Despite extensive research documenting attachment-loneliness associations, three critical gaps remain in the literature. First, while most studies focus on how attachment insecurity (anxiety and avoidance) contributes to different types of loneliness, fewer investigations examine the specific protective mechanisms through which attachment security operates. Understanding these mechanisms is crucial because they represent potentially modifiable pathways for intervention. Second, the role of self-disclosure quality as a proximal behavioral pathway linking attachment security to reduced loneliness has received limited empirical attention, despite strong theoretical rationale. Third, although social support is known to mediate attachment-loneliness relationships, the sequential processes through which disclosure behaviors mobilize social support resources remain underexplored.

The integration of attachment security, self-disclosure quality, and perceived social support into a comprehensive theoretical framework requires consideration of their sequential relationships and mechanisms through which they collectively influence loneliness outcomes. Serial mediation models provide a methodological approach for examining complex causal chains where multiple mediating variables operate in sequence (Hayes, 2022). This approach proves particularly appropriate for understanding psychological processes that unfold over time through interconnected behavioral and environmental mechanisms. The theoretical rationale draws from multiple complementary perspectives. Social cognitive theory emphasizes reciprocal interactions between personal characteristics, behavioral patterns, and environmental responses (Bandura, 1991). Attachment theory provides the foundation for understanding how internal working models translate into interpersonal behaviors, while social exchange theory illuminates how behavioral investments generate environmental returns in the form of social support (Blau, 2017). The integration suggests a causal sequence beginning with individual characteristics (attachment security), progressing through behavioral expressions (self-disclosure quality), generating environmental responses (perceived social support), and ultimately influencing psychological outcomes (loneliness).

The current study addresses these gaps by proposing and testing a serial mediation model in which attachment security reduces loneliness through two sequential pathways: enhanced self-disclosure quality, which in turn facilitates greater perceived social support. This model advances theoretical understanding by identifying specific behavioral (disclosure quality) and environmental (social support) mechanisms that explain how attachment security confers protection against loneliness in college students.

Based on this theoretical framework, four specific hypotheses guide the current investigation:

Hypothesis 1 (H1): Attachment security will demonstrate a significant negative direct effect on loneliness, such that individuals with higher attachment security will report lower levels of loneliness.
Hypothesis 2 (H2): Self-disclosure quality will significantly mediate the relationship between attachment security and loneliness, such that attachment security will positively predict self-disclosure quality, which will in turn negatively predict loneliness.
Hypothesis 3 (H3): Perceived social support will significantly mediate the relationship between attachment security and loneliness, such that attachment security will positively predict perceived social support, which will in turn negatively predict loneliness.
Hypothesis 4 (H4): Self-disclosure quality and perceived social support will sequentially mediate the relationship between attachment security and loneliness, such that attachment security will positively predict self-disclosure quality, which will positively predict perceived social support, which will in turn negatively predict loneliness.

**FIGURE 1:**
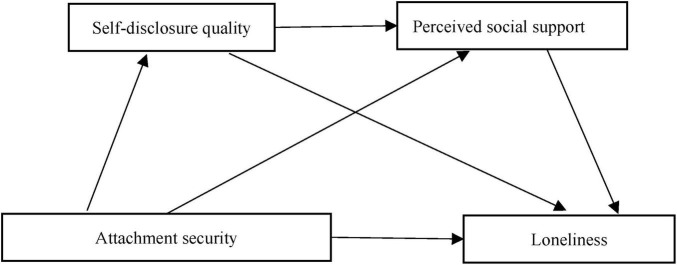
Theoretical model of serial mediation pathways.

The proposed model permits examination of the relative contributions of different pathways, providing insights into the primary mechanisms through which attachment security influences loneliness. This investigation contributes to attachment theory by illuminating specific behavioral and environmental mechanisms through which internal working models influence psychological outcomes, while advancing understanding of college student mental health by identifying potentially modifiable pathways for prevention and intervention efforts. As shown in Figure 1, theoretical model of serial mediation pathways linking attachment security to loneliness through self-disclosure quality and perceived social support. The model depicts direct pathways from attachment security to loneliness, simple mediation pathways through self-disclosure quality and perceived social support separately, and the complete serial mediation pathway through both mediators sequentially.

## 2 Materials and methods

### 2.1 Participants and research design

#### 2.1.1 Sampling procedure and participant recruitment

A stratified random sampling design was employed to recruit participants from five comprehensive universities across Sichuan Province, China, to ensure representativeness and external validity of findings. Within each university, stratified random sampling was conducted based on academic year and major field of study to achieve adequate representation across educational levels and academic disciplines. The initial sampling frame included 1,200 potential participants identified through university registrar databases. From this pool, 1,150 students agreed to participate, yielding a response rate of 95.8%. After excluding cases with incomplete data (*n* = 52), the final analytical sample comprised 1,098 college students, representing a 91.5% completion rate.

#### 2.1.2 Demographic characteristics

The final sample of 1,098 participants ranged in age from 17 to 25 years (*M* = 20.15, SD = 1.28). The sample included 910 female participants (82.9%) and 188 male participants (17.1%). This gender distribution reflects the enrollment patterns in the participating universities, where female students comprised approximately 80%–85% of the undergraduate population in the sampled social sciences and humanities programs during the 2024–2025 academic year. While this represents the actual demographic composition of our target population, we acknowledge this gender imbalance as a limitation that may affect generalizability, particularly given documented gender differences in attachment processes and loneliness experiences. The academic year distribution included 448 first-year students (40.8%), 355 third-year students (32.3%), 276 second-year students (25.1%), and 19 fourth-year students (1.7%). Family structure analysis revealed that 508 participants (46.3%) had one sibling, 235 participants (21.4%) were only children, and 355 participants (32.3%) had two or more siblings.

#### 2.1.3 Sample size adequacy and inclusion criteria

Power analysis for serial mediation using PROCESS Model 6 indicated that a minimum sample size of 550 participants would be required to detect small-to-medium indirect effects (*d* = 0.20) with 80% power at α = 0.05 (Fritz and MacKinnon, 2007). The achieved sample size of 1,098 participants provided substantial power (>0.99) for detecting hypothesized mediation effects. Inclusion criteria required participants to be currently enrolled undergraduate students aged 17–25 years with sufficient Chinese language proficiency to complete self-report measures. Exclusion criteria included current psychological counseling for severe mental health conditions and inability to provide informed consent.

#### 2.1.4 Ethical considerations and data collection procedures

Data collection was conducted between March and May 2025, following the complete stabilization of COVID-19 restrictions in China. This timing allowed for assessment of attachment-loneliness relationships during a period of fully resumed in-person educational activities and normal social interactions. The study received approval from the Ethics Review Committee of the Sichuan Psychological Association. All participants provided written informed consent after receiving detailed information about study purposes, procedures, and data confidentiality protections. Participants were explicitly informed of their right to withdraw from the study or delete their data at any time without penalty. Data collection was conducted through secure online survey platforms with encryption protocols^1^. Participants completed measures in a fixed order to minimize potential order effects, with completion time typically ranging from 25 to 35 min.

### 2.2 Measurement instruments

All instruments used in this study are publicly available for academic research purposes. Validated Chinese versions were employed for all measures to ensure cultural appropriateness and linguistic validity.

#### 2.2.1 Adult attachment scale - closeness dimension

Attachment security was assessed using the closeness dimension of the Adult Attachment Scale (Collins and Read, 1990), which measures general comfort with closeness and intimacy in close relationships rather than relationship-specific attachment patterns. This approach captures broad attachment orientation toward intimate relationships across different relationship types (romantic, friendship, family). The closeness dimension consists of 6 items rated on a five-point Likert scale ranging from 1 (not at all characteristic of me) to 5 (very characteristic of me). Sample items include “I find it easy to get close to others” and “I am comfortable depending on others.” Higher scores indicate greater comfort with closeness and intimacy, reflecting attachment security. The Chinese version has been validated in multiple studies and has demonstrated good psychometric properties (Wei et al., 2007). In the current study, the closeness dimension demonstrated good internal consistency (α = 0.85).

#### 2.2.2 Self-disclosure quality index

Self-disclosure quality was measured using a comprehensive index combining depth and honesty dimensions, assessed through validated Chinese versions of established instruments. The depth dimension was assessed using seven items from the Chinese version of the Self-Disclosure Index-Revised (Miller et al., 1983), measuring the personal significance and intimacy level of disclosed information. The honesty dimension was assessed using five items from the Self-Disclosure Honesty Scale (Wheeless and Grotz, 1977), evaluating the accuracy and authenticity of disclosed information. The Chinese adaptation and validation of these scales was conducted by Chen (2020). Both dimensions use five-point Likert scales ranging from 1 (strongly disagree) to 5 (strongly agree). The composite quality index was created by standardizing both dimension scores and computing their average, consistent with theoretical frameworks emphasizing quality over quantity (Reis and Shaver, 1988). In the current study, the composite self-disclosure quality index demonstrated excellent internal consistency (α = 0.84), with individual dimensions showing good reliability (depth: α = 0.87; honesty: α = 0.82).

#### 2.2.3 Multidimensional scale of perceived social support

Perceived social support was assessed using the Chinese version of the Multidimensional Scale of Perceived Social Support (MSPSS; Zimet et al., 1988), a widely validated 12-item instrument measuring perceived adequacy of social support from three sources: family (4 items), friends (4 items), and significant others (4 items). The scale employs a seven-point Likert response format ranging from 1 (very strongly disagree) to 7 (very strongly agree). Sample items include “My family really tries to help me” (family support), “I can count on my friends when things go wrong” (friends support), and “There is a special person with whom I can share my joys and sorrows” (significant others support). The Chinese version has been extensively validated and has consistently demonstrated strong psychometric properties in Chinese populations (Chou, 2000). In the current study, the MSPSS total score demonstrated excellent internal consistency (α = 0.94), with individual subscales showing good reliability: family support (α = 0.89), friends support (α = 0.88), and significant others support (α = 0.90).

#### 2.2.4 UCLA loneliness scale version 3

Loneliness was measured using the Chinese version of the UCLA Loneliness Scale Version 3 (Russell, 1996), a 20-item instrument assessing subjective feelings of loneliness and social isolation. The scale employs a four-point Likert response format ranging from 1 (never) to 4 (often). Sample items include “I feel left out” and “I feel isolated from others” (positively keyed items) and “I feel in tune with the people around me” and “I can find companionship when I want it” (negatively keyed items). The scale has demonstrated exceptional psychometric properties with internal consistency coefficients consistently exceeding.90 (Russell, 1996). The Chinese version has been extensively validated and has confirmed the factor structure and measurement invariance of the scale in Chinese populations (Wu and Yao, 2008). In the current study, the UCLA Loneliness Scale demonstrated excellent internal consistency (α = 0.91).

### 2.3 Statistical analysis strategy

Statistical analyses were conducted using SPSS 25.0 and PROCESS 4.0. Preliminary analyses included comprehensive data screening procedures to ensure statistical assumptions were met. Descriptive statistics for all study variables and bivariate correlation analyses were conducted to examine relationships among variables. Common method bias was assessed using Harman’s single-factor test, with the first factor accounting for 28.5% of total variance, well below the 40% threshold, indicating that common method bias was not a significant concern. Multicollinearity was assessed using variance inflation factors (VIF), with all values below 4.0, indicating acceptable levels of multicollinearity. Serial mediation analysis was conducted using PROCESS Model 6 (Hayes, 2022), which simultaneously examines multiple indirect pathways while providing rigorous statistical testing procedures. This approach offers advantages over traditional mediation procedures by: (a) directly testing indirect effects without requiring significant zero-order correlations, (b) using bootstrap confidence interval estimation that does not rely on normal distribution assumptions, and (c) simultaneously examining multiple mediators to control for shared variance. Model 6 specifically tests three indirect pathways: attachment security → self-disclosure quality → loneliness, attachment security → perceived social support → loneliness, and attachment security → self-disclosure quality → perceived social support → loneliness.

Bias-corrected bootstrap confidence intervals were computed using 5,000 bootstrap resamples to provide robust significance testing for indirect effects (Preacher and Hayes, 2008). The bootstrap approach generates empirical sampling distributions by repeatedly resampling from the original dataset, calculating indirect effects for each resample, and constructing confidence intervals from the resulting distribution. This method does not assume normality of the indirect effect sampling distribution and provides equivalent statistical power to traditional significance testing while offering more informative effect size information. The analysis strategy included systematic decomposition of total effects into direct and indirect components, with further partitioning of total indirect effects across specific pathways. Relative importance indices were calculated by expressing each specific indirect effect as a percentage of the total indirect effect, enabling comparison of pathway contributions. All continuous variables were mean-centered prior to analysis to facilitate interpretation of regression coefficients. Statistical significance was evaluated using α = 0.05 for all tests.

## 3 Results

### 3.1 Preliminary and descriptive analyses

Prior to conducting the serial mediation analysis, comprehensive data screening procedures were implemented to ensure statistical assumptions were met. Missing data analysis revealed complete data for all 1,098 participants across the four core study variables, eliminating concerns about listwise deletion bias (Schafer and Graham, 2002). Univariate outliers were assessed using standardized z-scores exceeding ±3.29, representing a conservative threshold corresponding to *p* < 0.001 for a two-tailed test, which provides a rigorous criterion for identifying extreme values while minimizing the risk of inappropriately removing valid data points (Tabachnick and Fidell, 2019). No cases met this criterion for removal. Multivariate outliers were evaluated through Mahalanobis distance calculations, with no observations exceeding the critical chi-square value at *p* < 0.001. Normality assumptions were examined through Shapiro-Wilk tests and visual inspection of histograms and Q-Q plots. While statistical tests indicated significant deviations from perfect normality due to the large sample size, skewness and kurtosis values for all variables remained within acceptable ranges (±2.0), supporting the use of parametric procedures (Tabachnick and Fidell, 2019). Linearity was confirmed through examination of bivariate scatterplots, revealing appropriate linear relationships among study variables. Homoscedasticity was verified through residual plot inspection, showing consistent error variance across predicted values.

As shown in Table 1, the correlation matrix revealed significant bivariate associations among all study variables in theoretically expected directions. Perceived social support demonstrated the strongest direct correlation with loneliness (*r* = −0.569, *p* < 0.001), while attachment security showed the second strongest association (*r* = −0.547, *p* < 0.001). Self-disclosure quality also showed a significant negative correlation with loneliness (*r* = −0.350, *p* < 0.001). These preliminary correlations provided initial support for the hypothesized mediation pathways, with perceived social support emerging as the most robust predictor of reduced loneliness. Multicollinearity diagnostics yielded variance inflation factors below 4.0 for all predictors, indicating acceptable levels of multicollinearity for regression analysis (Hair et al., 2019). Descriptive statistics revealed that participants reported moderate levels across, all measured constructs with distributions approximating normality.

**TABLE 1:** Descriptive statistics and intercorrelations among study variables.

Variable	*M*	SD	Skewness	Kurtosis	1	2	3
1. AS	19.213	2.812	−0.150	−0.080	–	–	–
2. SDQ	61.532	7.569	−0.120	0.210	0.294^***^	–	–
3. PSS	56.217	12.662	−0.260	−0.150	0.470^***^	0.424^***^	–
4. LON	44.841	8.810	0.180	−0.230	−0.547^***^	−0.349^***^	−0.569^***^

*N* = 1,098. AS, attachment security; SDQ, self-disclosure quality; PSS, perceived social support; LON, loneliness.

^**^P < 0.01,

^***^P < 0.001.

### 3.2 Serial mediation model analysis

The serial mediation analysis was conducted using PROCESS Model 6 (Hayes, 2022) with attachment security as the independent variable (X), self-disclosure quality as the first mediator (M1), perceived social support as the second mediator (M2), and loneliness as the dependent variable (Y). Bootstrap confidence intervals were generated using 5,000 resamples with bias correction to account for potential non-normality in the sampling distribution of indirect effects.

The results in Table 2 provide strong support for all hypothesized pathways. The first regression equation predicting self-disclosure quality from attachment security yielded significant results, F(1, 1096) = 104.162, *p* < 0.001, R^2^ = 0.087. Attachment security significantly predicted self-disclosure quality (*B* = 0.791, SE = 0.078, *p* < 0.001), accounting for 8.7% of its variance, supporting H2. The second regression equation predicting perceived social support from both attachment security and self-disclosure quality demonstrated substantial predictive power, F(2, 1095) = 246.748, *p* < 0.001, R^2^ = 0.310. Both attachment security (B = 1.699, SE = 0.118, *p* < 0.001) and self-disclosure quality (*B* = 0.523, SE = 0.044, *p* < 0.001) emerged as significant predictors, collectively explaining 31.0% of perceived social support variance. The final regression equation predicting loneliness from all three predictors achieved strong model performance, F(3, 1094) = 277.678, *p* < 0.001, R^2^ = 0.432. All predictors significantly contributed to loneliness prediction: attachment security (*B* = −1.094, SE = 0.081, *p* < 0.001), self-disclosure quality (*B* = −0.107, SE = 0.030, *p* < 0.001), and perceived social support (*B* = −0.255, SE = 0.019, *p* < 0.001). The complete model explained 43.2% of loneliness variance, representing a large effect size (Cohen, 1988), supporting H1 and H3.

**TABLE 2:** Path coefficients and model parameters for serial mediation analysis.

Outcome variable	Predictor	*B*	SE	*t*	*P*	95% CI	β
SDQ	Constant	46.328	1.505	30.785	<0.001	[43.380, 49.280]	–
(R^2^ = 0.087)	AS	0.791	0.078	10.206	<0.001	[0.640, 0.940]	0.294
PSS	Constant	−8.649	2.988	−2.895	0.004	[−14.510, −2.790]	–
(R^2^ = 0.310)	AS	1.699	0.118	14.403	<0.001	[1.470, 1.930]	0.378
	SDQ	0.523	0.044	11.926	<0.001	[0.440, 0.610]	0.313
LON	Constant	86.752	1.896	45.757	<0.001	[83.030, 90.470]	–
(R^2^ = 0.432)	AS	−1.094	0.081	−13.449	<0.001	[−1.250, −0.930]	−0.349
	SDQ	−0.107	0.030	−3.619	<0.001	[−0.160, −0.050]	−0.092
	PSS	−0.255	0.019	−13.353	<0.001	[−0.290, −0.220]	−0.366

*N* = 1,098. AS, attachment security; SDQ, self-disclosure quality; PSS, perceived social support; LON, loneliness. All predictors mean-centered. Bootstrap confidence intervals based on 5,000 samples.

The total effect of attachment security on loneliness was significant (*B* = −1.717, SE = 0.079, *p* < 0.001), indicating that each unit increase in attachment security corresponded to a 1.720-point decrease in loneliness scores. The direct effect remained significant after inclusion of both mediators (*B* = −1.094, SE = 0.081, *p* < 0.001), suggesting partial mediation through the proposed pathways.

**FIGURE 2:**
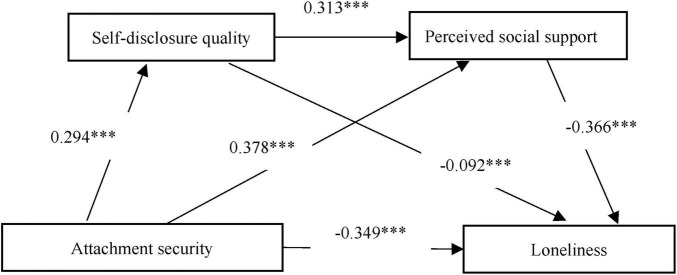
Serial mediation model with standardized path coefficients. ***P < 0.001.

The Figure 2 displays the tested serial mediation model linking attachment security to loneliness through self-disclosure quality and perceived social support. Standardized path coefficients (β) are shown along each pathway, with all paths significant at *p* < 0.001. The model demonstrates the direct pathway from attachment security to loneliness (β = −0.349), as well as the indirect pathways through self-disclosure quality (β = 0.294 → β = −0.092) and perceived social support (β = 0.378 → β = −0.366), and the complete serial pathway (β = 0.294 → β = 0.313 → β = −0.366). R^2^ values indicate the proportion of variance explained in each endogenous variable: self-disclosure quality (8.7%), perceived social support (31.0%), and loneliness (43.2%).

### 3.3 Multiple pathway decomposition

The pathway decomposition results are presented in Table 3. The serial mediation model yielded significant total indirect effects (*B* = −0.620, SE = 0.050), with bootstrap confidence intervals excluding zero [95% CI: −0.730, −0.520]. This total indirect effect was decomposed into three specific pathways, each tested for statistical significance through bias-corrected bootstrap procedures.

**TABLE 3:** Indirect effects decomposition and pathway contributions.

Pathway	*B*	BootSE	95% CI	Percentage
Total indirect effects	−0.623	0.054	[−0.730, −0.519]	100.00%
Ind1: AS → SDQ → LON	−0.084	0.028	[−0.140, −0.032]	13.60%
Ind2: AS → PSS → LON	−0.433	0.043	[−0.521, −0.350]	69.50%
Ind3: AS → SDQ → PSS → LON	−0.110	0.019	[−0.146, −0.072]	16.90%

AS, attachment security; SDQ, self-disclosure quality; PSS, perceived social support; LON, loneliness. Bootstrap confidence intervals based on 5,000 samples.

The pathway decomposition results are presented in Table 3. The serial mediation model yielded significant total indirect effects (*B* = −0.623, SE = 0.054), with bootstrap confidence intervals excluding zero [95% CI: −0.730, −0.519]. This total indirect effect was decomposed into three specific pathways, each tested for statistical significance through bias-corrected bootstrap procedures. Pathway analysis in Table 3 revealed differential contributions among the three indirect effects, providing strong support for H4. The strongest mediation occurred through the direct pathway from attachment security to perceived social support to loneliness (Ind2: *B* = −0.433), accounting for 69.5% of the total indirect effect. The complete serial mediation pathway (Ind3: *B* = −0.106) contributed 16.9% of the total indirect effect, while the simple mediation through self-disclosure quality alone (Ind1: *B* = −0.084) accounted for 13.6% of the total indirect effect. Contrast analyses examined the relative magnitudes of the three indirect pathways. The difference between Ind2 and Ind1 was significant (difference = −0.349, 95% CI: [−0.434, −0.264]), indicating that the pathway through perceived social support alone was significantly stronger than the pathway through self-disclosure quality alone. Similarly, the difference between Ind2 and Ind3 was significant (difference = −0.323, 95% CI: [−0.398, −0.248]), demonstrating the superiority of the direct social support pathway over the complete serial pathway. The difference between Ind1 and Ind3 was also significant (difference = 0.026, 95% CI: [0.003, 0.049]), with the serial pathway showing slightly stronger effects than the simple disclosure pathway.

## 4 Discussion

### 4.1 Summary of major findings

The present study successfully validated a serial mediation model examining the pathways through which attachment security relates to loneliness among college students. The findings provide robust empirical support for the hypothesized chain mediation involving self-disclosure quality and perceived social support as sequential mediators. The model demonstrated substantial explanatory power, accounting for 43.2% of the variance in loneliness scores, representing a large effect size that underscores the theoretical and practical significance of these protective mechanisms (Cohen, 1988).

The pathway decomposition revealed differential contributions across the three indirect effects, providing novel insights into the relative importance of various mechanisms. The direct pathway from attachment security through perceived social support to loneliness emerged as the dominant mediating mechanism, accounting for 69.50% of the total indirect effect, emphasizing the central role of social support perception as a proximal predictor of psychological adjustment (Gariépy et al., 2016). The complete serial mediation pathway contributed 16.90% of the total indirect effect, while the direct pathway through self-disclosure quality alone accounted for 13.60%, suggesting that disclosure behaviors may primarily influence loneliness through their capacity to mobilize social support rather than through direct effects.

These results provide comprehensive support for all four hypothesized relationships: H1 (attachment security negatively predicts loneliness), H2 (self-disclosure quality mediates the attachment-loneliness relationship), H3 (perceived social support mediates the attachment-loneliness relationship), and H4 (self-disclosure quality and perceived social support sequentially mediate the attachment-loneliness relationship). The successful validation of this complex theoretical model represents a significant advancement in understanding the mechanisms underlying psychological adjustment in college populations.

### 4.2 Theoretical implications and contributions

Our findings extend previous attachment-loneliness research in several important ways, positioning our work within recent theoretical advances in loneliness research. While prior studies have predominantly examined how attachment insecurity (anxiety and avoidance) contributes to different types of subjective disconnection, our research demonstrates the protective mechanisms through which attachment security operates. Recent theoretical frameworks distinguish between emotional loneliness (perceived absence of close relationships), social loneliness (perceived absence of broader social networks), and existential isolation (perceived absence of shared perceptions and experiences) (Helm, 2025). Previous research has shown that anxious attachment relates more strongly to emotional loneliness, while avoidant attachment correlates with social loneliness and existential isolation (Helm et al., 2020; Bergman et al., 2024). Our contribution lies in examining how attachment security provides broad protection against overall loneliness by facilitating high-quality interpersonal behaviors that mobilize social support resources.

The current findings extend attachment theory by illuminating specific behavioral and environmental pathways through which internal working models relate to psychological outcomes. The significant pathway from attachment security to self-disclosure quality suggests an association between secure internal working models and adaptive interpersonal behaviors, supporting theoretical propositions about the behavioral expression of attachment orientations (Zhang et al., 2022). Furthermore, the identification of multiple protective pathways supports nuanced theoretical frameworks recognizing the multifaceted nature of attachment security’s protective effects, consistent with contemporary attachment theorizing that emphasizes the dynamic and contextual nature of attachment processes (Duschinsky, 2020).

The results provide empirical support for theoretical frameworks emphasizing disclosure quality over quantity in determining relationship and adjustment outcomes. The sequential mediation pathway validates theoretical propositions that attachment security may enhance disclosure quality, which in turn facilitates social support mobilization (Gillath et al., 2010; Luo and Hancock, 2020). The dominant role of perceived social support in mediating the attachment–loneliness relationship provides strong validation for theories positioning social support as a central mechanism in psychological adjustment, and aligns with cognitive models arguing that perceived support is more influential than received/objective support (Benoit and DiTommaso, 2020; Grey et al., 2020).

These findings contribute to emerging theoretical frameworks recognizing the unique developmental challenges and protective mechanisms relevant to college student populations. The substantial variance explained by the model suggests that attachment security, disclosure quality, and social support represent core components of a comprehensive framework for understanding loneliness prevention in college populations, supporting developmental theories emphasizing the continued importance of attachment processes in emerging adulthood (Benoit and DiTommaso, 2020; Shaver and Mikulincer, 2005).

### 4.3 Clinical and educational applications

The pathway decomposition results provide guidance for developing targeted interventions addressing loneliness through multiple mechanisms. Given the dominant role of perceived social support (69.5% of indirect effects), interventions focusing on enhancing students’ capacity to recognize, seek, and effectively utilize available social resources may be prioritized. Cognitive-behavioral interventions targeting social support perception could include cognitive restructuring techniques to address negative biases in interpreting social interactions and behavioral activation strategies to increase engagement with potential support sources (Hickin et al., 2021). The significant contribution of the serial mediation pathway (16.9%) suggests that interventions combining attachment-focused approaches with disclosure skill training may provide additional benefits. Such interventions might focus on helping students recognize and modify maladaptive relationship patterns while simultaneously developing adaptive disclosure behaviors. The direct disclosure pathway (13.6%) indicates that targeted skills training in high-quality self-disclosure could provide meaningful benefits, with interventions emphasizing quality dimensions rather than frequency of personal sharing.

The validated model provides a framework for systematic screening and early identification of students at risk for loneliness and related mental health difficulties. Assessment protocols could evaluate attachment security levels, disclosure quality behaviors, and perceived social support to identify students most likely to benefit from preventive interventions (Benoit and DiTommaso, 2020; Luo and Hancock, 2020). The transition to college represents an optimal intervention window when relationship patterns are being established and social networks are forming (Carcedo et al., 2023). The integration of mental health promotion with existing campus programming offers opportunities for population-level intervention. Residence hall programming, student organization activities, and academic support services could incorporate components addressing attachment security, disclosure skill development, and social support enhancement, normalizing mental health promotion while reaching students who might not otherwise seek psychological services (Käll et al., 2020).

### 4.4 Limitations and future research

Several limitations should be acknowledged when interpreting these findings. The cross-sectional design limits the ability to make definitive causal inferences, as alternative explanations cannot be ruled out—students with lower loneliness might develop enhanced attachment security as a result rather than antecedent (Maxwell and Cole, 2007). A significant limitation is our measurement of overall loneliness rather than distinguishing between different forms of subjective disconnection. Recent advances have distinguished between emotional loneliness, social loneliness, and existential isolation, each associated with different attachment patterns (Walsh et al., 2025; Hemberg et al., 2022). Critical reviews emphasize that emotional loneliness appears to be a more severe typology that overlaps greatly with pathology, whereas social loneliness is more reflective of deficits in social connectedness and social support (Walsh et al., 2025). Research shows that existential isolation relates differently to attachment orientations compared to traditional loneliness measures (Galgali et al., 2023; Garnow et al., 2022), suggesting our focus on general loneliness may have obscured differential effects of attachment security on specific disconnection types. Additional limitations include reliance on self-report measures raising common method variance concerns, the predominantly female sample (82.9%) limiting generalizability given documented gender differences in attachment and disclosure processes (Cross and Madson, 1997), and the Chinese cultural context raising questions about cross-cultural generalizability of pathway importance. Cultural and temporal factors significantly influence loneliness experiences (Luhmann et al., 2023), further emphasizing the need for cross-cultural validation of our findings (van IJzendoorn and Sagi-Schwartz, 2008; Chen, 1995).

Critical next steps involve implementing longitudinal designs to establish temporal precedence and examine how attachment security, disclosure behaviors, and social support evolve over time, particularly important given that large-scale longitudinal evidence shows loneliness peaks during emerging adulthood before declining into midlife (Graham et al., 2024). Future research should examine more precise mechanisms using neuroscience methods to understand biological bases of attachment security effects (Eisenberger and Cole, 2012), conduct cross-cultural studies to establish universality versus cultural specificity of findings, and develop targeted interventions based on identified pathways. Most importantly, future studies should utilize multidimensional loneliness measures to provide more nuanced understanding of how attachment security operates across different forms of subjective disconnection. Randomized controlled trials comparing pathway-focused interventions could establish causal effects and identify optimal approaches, with dismantling studies examining intervention component contributions.

## 5 Conclusion

This investigation successfully validated a comprehensive serial mediation model elucidating the mechanisms through which attachment security relates to loneliness among college students. The findings demonstrate that perceived social support serves as the primary mediating pathway, while the complete behavioral-environmental sequence through self-disclosure quality and social support represents a significant secondary mechanism. The identification of social support perception as the dominant mediating mechanism suggests that interventions should prioritize enhancing students’ capacity to recognize, access, and utilize available support resources, while targeted skill-building programs addressing high-quality self-disclosure behaviors may provide additional protective benefits. These findings call for continued research examining the complex interplay of individual characteristics, interpersonal behaviors, and environmental resources in promoting psychological adjustment. Future investigations should explore the temporal dynamics of these protective mechanisms and their effectiveness across diverse student populations, ultimately informing evidence-based approaches to fostering resilience and wellbeing during critical developmental transitions in higher education contexts.

## Data Availability

The raw data supporting the conclusions of this article will be made available by the authors, without undue reservation.

## Footnotes

1
https://www.wjx.cn/
